# Retrospective Descriptive Case Series on the Use of AMCOP^®^ Elastodontic Appliance in Growing Patients with Class III Malocclusion

**DOI:** 10.3390/bioengineering13050504

**Published:** 2026-04-26

**Authors:** Angelo Michele Inchingolo, Alessio Danilo Inchingolo, Filippo Cardarelli, Francesco Inchingolo, Daniela Di Venere, Elisabetta de Ruvo, Laura Ferrante, Grazia Marinelli, Andrea Palermo, Gianna Dipalma

**Affiliations:** 1 Department of Interdisciplinary Medicine, University of Bari “Aldo Moro”, 70121 Bari, Italy; angelomichele.inchingolo@uniba.it (A.M.I.); alessiodanilo.inchingolo@uniba.it (A.D.I.); drfilippocardarelli@libero.it (F.C.); daniela.divenere@uniba.it (D.D.V.); studio.deruvo@libero.it (E.d.R.); lauraferrante79@virgilio.it (L.F.); grazia.marinelli@uniba.it (G.M.); gianna.dipalma@uniba.it (G.D.); 2 Department of Experimental Medicine, University of Salento, 73100 Lecce, Italy; andrea.palermo@unisalento.it

**Keywords:** interceptive orthodontics, third classes, elastodontics, elastodontic therapy, elastodontic appliances

## Abstract

Background: This retrospective case series evaluated the descriptive clinical observations of the bio-activator AMCOP^®^ TC in the treatment of growing patients with Class III dento-skeletal malocclusion. In recent years, elastodontic appliances have been introduced as an evolution of conventional functional appliances. Elastodontic therapy could be an excellent therapeutic alternative in the early treatment of patients with Class III dento-skeletal malocclusion. Aim: This retrospective experimental study evaluated the descriptive clinical observations of the bio-activator AMCOP^®^ TC in the treatment of patients with Class III dento-skeletal malocclusion and described four clinical cases. Materials and methods: The study included 11 subjects (5 males and 6 females, aged between 3 and 12 years) treated with the AMCOP^®^ TC bio-activator for Class III dento-skeletal malocclusion. Patients used the AMCOP^®^ TC device for two hours in the afternoon and all night for 6–8 months and then only at night. For each patient, cephalometric analyses were performed on latero-lateral teleradiographs both at the beginning of treatment (T0) and at the end of treatment (T1). Analyses were performed using DeltaDent^®^ software. Conclusions: Cephalometric observations between T0 and T1 showed changes in sagittal relationship parameters, including ANB values; however, these findings should be interpreted cautiously. Elastodontic therapy with an AMCOP^®^ TC appliance improved the correction of a Class III dento-skeletal malocclusion and postural restoration of the first cervical vertebrae. Although further studies are needed, AMCOP^®^ TC bio-activators may be considered a possible interceptive treatment approach in selected growing patients; however, the present findings should be interpreted with caution. Findings should be considered preliminary and interpreted with caution.

## 1. Introduction

Class III dento-skeletal malocclusion, which remains a therapeutic challenge today, is characterised by a sagittal alteration of the normal relationships between the maxillary bones [1,2,3]. In particular, the lower jaw is advanced in relation to the upper jaw; there may also be a reversal of the physiological relationship between the incisors (anterior crossbite) [4,5,6]. When the change affects the dental component, it is referred to as dental Class III; when the change affects the skeletal structures of the upper and/or lower jaw, it is referred to as skeletal Class III [7,8,9].

In the early 1990s, Angle described a Class III dento-skeletal malocclusion as one in which the mesio-vestibular cusp of the maxillary first molar occludes distal to the mesio-vestibular sulcus of the mandibular first molar [10,11,12]. A skeletal Class III malocclusion may be caused by overdevelopment of the maxilla and/or overdevelopment of the mandible [13,14,15].

The diagnosis of dental Class III can be made by clinical examination, whereas the diagnosis of skeletal Class III requires cephalometric analysis on teleradiography in latero-lateral projection [7,16,17].

The width of the cephalometric angle ANB, which represents the sagittal discrepancy between the maxilla and the mandible, is assessed [18,19,20]. If ANB is between 0° and 4°, it is a Class I malocclusion; if ANB is >4°, it is a Class II malocclusion; if ANB is <0°, it is a Class III malocclusion [21,22,23]. Cephalometric point A (Downs’ or sub-spinal point A) is the most depressed point of the anterior maxilla, cephalometric point N (nasion) is the most anterior point of the naso-frontal suture, and cephalometric point B (Downs’ or supra-mental point B) is the most depressed point of the anterior mandibular symphysis [24,25,26].

The aetiology of Class III dento-skeletal malocclusion is multifactorial [27,28,29]. In 1980, Ferro defined the aetiological factors as genetic and epigenetic or environmental factors [30,31,32]. With regard to the genetic component, Class III manifestations in the same family suggest a dominant mode of inheritance. Regarding epigenetic or environmental factors, low tongue position, which may be due to oral breathing, swallowing disorders, short frenulum, and labio-palatal schisis, causes overdevelopment of the maxilla and overdevelopment of the mandible [33,34,35].

A Class III dento-skeletal malocclusion can be associated with a normal bite, a deep bite or an open bite [36,37,38]. In Class III normo-bite, which has a favourable prognosis, the vertical dimension is normal. In Class III deep bite, which has a favourable prognosis, there is a reduction in the vertical dimension. In Class III open bite, which has an unfavourable prognosis, the vertical dimension is increased [39,40,41].

Clinically, typical signs of Class III are advancement of the lower dental arch relative to the upper, a concave profile with protrusion of the lower lip (lower procheilia) and/or retrusion of the upper lip (upper retrocheilia) [42,43,44].

Class III may also be associated with sleep apnoea, joint pain, phonation disorders, back pain, recurrent headaches, coughing, sinusitis, and gastro-oesophageal reflux; a condition that may also affect psychological status [45,46,47].

Orthopaedic therapy, which uses the growth potential of the jaw bones is one of the main treatment options [48,49,50].

The aim of this retrospective experimental study was to evaluate the descriptive clinical observations of elastodontic therapy with the AMCOP^®^ TC bio-activator in growing patients with Class III dento-skeletal disorders.

**Figure 1:**
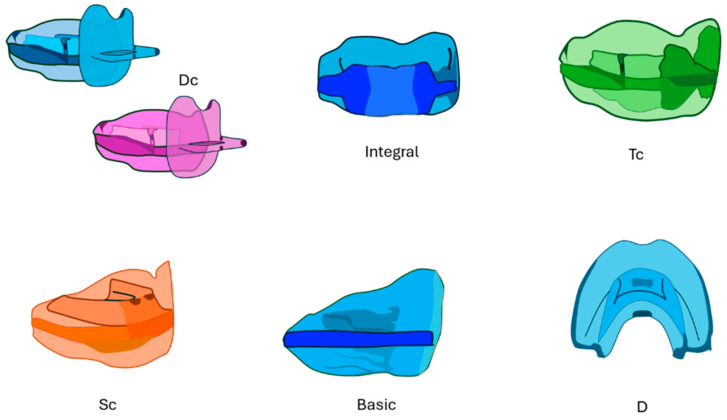
AMCOP^®^ elastodontic devices on the market.

The Multifunctional Cranio-Occluso-Postural Harmoniser (AMCOP^®^) has the function of harmoniously remodelling the dento-cranio-facial structures, correcting various functional problems through neuro-muscular re-education, and acting on the relationship between the skull and the jaw bones, as well as between occlusion and posture, by acting on the first cervical vertebrae (Figure 1) [51,52,53].

The AMCOP^®^ bio-activators consist of two flanges, one vestibular and one lingual, which define a central area of equilibrium between the force of the tongue (centrifugal force) and the force of the cheek and lip (centripetal forces) in which the teeth are positioned [54,55,56]. The vestibular flange performs the function of a lip bumper; in fact, it pulls away the perioral muscles and provides proprioceptive stimulation of the bone matrix [57,58,59]. It involves both arches and has an orthopaedic effect on the jaw bones transversely, sagittally, vertically, and torsionally [60,61,62]. The AMCOP^®^ bio-activators also consist of a lingual ramp and a tongue button, which guide the tongue towards the palate and help to restore the correct lingual position and function [63,64,65]. There are also drains to avoid interference with the frenula and retro-incisal papilla, and two concavities at the level of the canine bumps [66,67,68].

The size of the device is chosen according to the transverse distance between the vestibular cusps of the upper sixth [69,70].

The AMCOP^®^ bio-activators are made of a highly elastic, thermoplastic, heat-activated polymer/elastomer mixture, available in two different Shore hardness grades (51 and 60), depending on the surgical requirements [71,72,73,74]. It can be expanded by immersion in hot water (70 °C) for 30 s and then in cold water [75,76,77].

The AMCOP^®^ bio-activators for deciduous dentition patients are particularly suitable for the treatment of children with maxillary hypodevelopment and open bite due to the low lingual position and/or prolonged dummy use. These devices are available in two models, D and DC, with or without a dummy handle [78,79,80].

The AMCOP^®^ bio-activators can be classified as first, second (SC) or third class (TC) devices [81,82,83]. First-class AMCOP^®^ are indicated for transversal and vertical disharmonies; they are suitable for deciduous, mixed, and permanent dentition [84,85,86]. They can have four different arch forms: F-form, for dolichocephals with ogival arches and narrow palates; S-form, for mesocephals with oval arches; OS-form, for mesocephals with square arches; and C-form, for brachycephals with wide, rounded arches and flat palates [87,88,89,90]. In addition, the occlusal plane can be integral, i.e., flat (suitable for patients with a normal bite), or basic, i.e., thicker in the anterior region (suitable for patients with a deep bite) [91,92,93,94].

SC AMCOP^®^, indicated for Class II malocclusions, consist of an anterior mandibular gliding plane; they control the growth of the upper jaw and correct the overjet by means of a superior incisal retro-inclination and an inferior incisal pro-inclination [95,96,97].

TC AMCOP^®^, indicated for Class III dento-skeletal malocclusions, stop the growth of the lower jaw and re-educate the tongue into a physiological position. In the case of an open bite, elastodontic therapy must be started within five years [98,99,100,101,102,103,104].

The present retrospective experimental study was conducted to evaluate the descriptive clinical observations of the AMCOP^®^ TC bio-activator device in the treatment of patients with Class III dento-skeletal malocclusions, describing four retrospective clinical cases.

In addition to the vertical pattern, treatment prognosis and appliance selection in Class III dento-skeletal malocclusion also depend on the severity and nature of the discrepancy. Elastodontic appliances such as AMCOP^®^ TC are mainly indicated as an interceptive approach in growing patients with mild Class III presentations, where dentoalveolar compensation and neuromuscular re-education can meaningfully contribute to sagittal correction. Favourable features for AMCOP^®^ TC therapy include a limited skeletal discrepancy (e.g., slightly negative or borderline ANB/Wits), a predominantly dentoalveolar component (anterior crossbite with incisor compensation), manageable arch-length discrepancy/crowding, and the absence of marked transverse constriction requiring rapid maxillary expansion as a primary step. Conversely, cases with severe skeletal Class III, pronounced mandibular prognathism, significant maxillary deficiency requiring protraction protocols, relevant arch-length deficiency, or unfavourable vertical patterns (especially open bite with increased vertical dimension) may show limited response to elastodontic mechanisms alone and often require alternative or adjunct orthopaedic approaches.

### Sample Selection

A sample of 11 subjects (5 males and 6 females, aged 3 to 12 years) treated with AMCOP^®^ TC bio-activator was enrolled from patients of the Orthodontic Department of the Bari Polyclinic and a private clinic.

Inclusion criteria:

- Skeletal Class III relationship at baseline, defined clinically and cephalometrically;
- Treatment with AMCOP^®^ TC appliance during the study period;
- Availability of complete pre-treatment and post-treatment clinical and radiographic records.

Exclusion criteria:

- Previous orthodontic treatment;
- Craniofacial syndromes or congenital anomalies;
- Incomplete records.

Informed consent was obtained from each patient. The following materials were collected: extraoral photographs, intraoral photographs, panoramic radiographs, and latero-lateral teleradiographs at the beginning of the treatment/observation period (T0) and at the end of treatment (T1).

Due to the exploratory nature of this retrospective case series, no formal sample size calculation was performed. The sample included all eligible patients treated with the AMCOP^®^ TC appliance during the study period.

Patients were retrospectively selected from those consecutively treated at the Orthodontic Department of the Bari Polyclinic and a private orthodontic clinic during the study period. No eligible patients meeting the inclusion criteria were excluded.

## 2. Materials and Methods

### 2.1. Treatment Protocol

The treatment plan included the use of the AMCOP^®^ TC bio-activator (Micerium S.p.A., Avegno, Genoa, Italy), which is indicated for the treatment of Class III dento-skeletal malocclusions in the primary, mixed, and permanent dentition. All clinical photographs and radiographs were obtained at baseline (T0) and at the end of treatment (T1). The average observation period between T0 and T1 was approximately 16–24 months, depending on the individual treatment duration.

**Figure 2:**
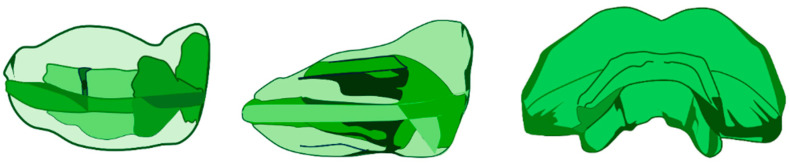
AMCOP^®^ TC bio-activator.

The AMCOP^®^ TC is a device with a maxillary sliding occlusal plane and a mandibular stop (Figure 2).

*AMCOP^®^ TC design and reactivation.*

The AMCOP^®^ TC is a preformed, one-piece elastodontic appliance engaging both arches, characterised by vestibular and lingual flanges (lip-bumper effect and neutral-zone guidance), a tongue button/lingual ramp to promote a physiological tongue posture, and a Class III-specific occlusal design with a maxillary sliding plane and mandibular stop. Appliance size was selected based on the transverse distance between the buccal cusps of the maxillary first molars. When additional transverse development was required, the appliance was reactivated by thermoplastic expansion (hot water ~70 °C for ~30 s), gently widened in the posterior segments, and then cooled in cold water to stabilise the new shape before intraoral fit verification. The appliances used in this study had a Shore hardness of approximately 60, as recommended for Class III treatment.

Patients wore the device for two hours in the afternoon and all night for 6–8 months and then only at night for the following months.

The following cephalometric parameters, SNA, SNB, and ANB were taken into consideration as well as the clinical occlusal improvement. The pre- and post- treatment cephalometric values were compared to evaluate the success of the orthodontic treatment. Compliance was monitored through parental report during follow-up visits.

*Biomechanical mechanism*

The AMCOP^®^ TC appliance acts through light elastic forces generated by the elastomeric material and through neuromuscular re-education. The vestibular shields reduce the centripetal pressure of the perioral muscles, favouring transverse development of the maxillary arch. The Class III occlusal configuration with a maxillary sliding plane and mandibular stop guides the mandible posteriorly during closure, producing a sagittal orthopaedic effect and encouraging dentoalveolar compensation. At the same time, the lingual ramp promotes tongue elevation toward the palate, contributing to functional rebalancing and stabilisation of the corrected occlusal relationship.

This study was approved by the Ethics Committee.

Prot. Number: 00152571

15/02/2023 JAOUCPG23ICOMETIP

Study number: 7593 Approved date 25 January 2023

U.O. di Odontostomatologia

CODE: EAP AS

Principal investigator: Prof. F. Inchingolo

U.O. di Odontostomatologia

University Polyclinic of Bari

### 2.2. Cephalometric Analysis

All radiographs were obtained with standardised head positioning and natural head posture using the same radiographic equipment. For each patient, cephalometric analyses were performed on latero-lateral teleradiographs at the beginning of treatment/observation period (T0) and at the end of treatment (T1). All cephalometric tracings were performed by the same operator using DeltaDent^®^ software (DeltaDent^®^, Lana, Italy), and measurements were repeated twice to minimise potential measurement error. The same reference cephalometric parameters were considered for all patients to allow a consistent comparison between pre- and post-treatment measurements. Descriptive comparisons between T0 and T1 cephalometric values were performed; no inferential statistical tests were applied due to the limited sample size.

## 3. Discussion

Class III dento-skeletal malocclusions represent a challenge for modern orthodontics as they are characterised by very complex therapeutic management. Functional or interceptive therapy is a valid treatment option for growing patients [105,106,107,108].

### 3.1. Traditional Functional Therapy

Functional orthodontics, which was introduced in Europe at the beginning of the 20th century, involves the use of various types of orthodontic devices [109,110,111]. It is indicated for the treatment of malocclusions in adolescents and has an orthopaedic effect on the bases of the jaws [112,113,114].

The ideal age for the use of functional appliances is generally late mixed dentition; however, interceptive treatment may also be initiated earlier in selected cases when functional or dentoalveolar factors are present [115,116,117].

Functional braces are mobile drop devices, i.e., they are designed to drop and encourage the patient to close the mouth so that they can be activated by the action of the tongue on the palate [118,119,120]. In this way, they act on neuromuscular tissues, deactivating non-physiological muscular forces and allowing the patient to re-establish a new occlusal balance [121,122,123].

Functional devices can be divided into activators and morphoconformers, depending on how they work [124,125,126,127]. Morphoconformers include the Fränkel function regulators, which modify the morphology of soft tissues, inactivating abnormal muscle forces and allowing skeletal structures to grow [128,129,130,131]. From a clinical point of view, the use of functional devices during the night and for a few hours (1–2 h) in the afternoon is indicated [132,133,134,135]. In fact, during the night, the device acts on involuntary circuits such as heartbeat, blinking, breathing, and swallowing, while in the afternoon, the involuntary stimulus becomes conscious at the level of the cerebral cortex [136,137,138,139].

However, such devices often cause discomfort to young patients, with a failure rate of up to 34% due to poor treatment compliance [140,141,142].

In order to increase patient compliance, fixed devices such as the Herbst, Forsus, and Powerscope have been introduced to treat Class II malocclusions [143,144,145,146].

Numerous authors have conducted studies regarding the possible treatments of a Class III dento-skeletal malocclusion in order to identify the most effective and to reduce the risk of recurrence of the dysgnathia.

The treatment of the transverse defect related to the upper jaw involves expanding the palate so that the jaw can be positioned correctly [147,148,149]. Palatal expanders are orthodontic devices that expand the upper jaw by acting on the medial palatine suture [150,151,152,153].

Maspero et al. treated patients with maxillary hypodevelopment with Rapid Palatal Expander (RPE) and with Transverse Sagittal Maxillary Expander (TSME). Both types of therapy provided transverse expansion of the upper jaw, with similar results regarding inter-molar distance, palatal depth, and palate length. However, TSME proved more effective in improving the perimeter of the maxillary arch [154,155,156,157].

Minase et al. compared patients with Class III dento-skeletal malocclusion treated with Reverse Twin Block with Lip Pads-Rapid Maxillary Expander (RTBLP-RME) and with Face Mask-Rapid Maxillary Expander (FM-RME). After 9 months, both types of therapy significantly improved the position of the upper jaw and mandible; RTBLP-RME achieved greater skeletal improvements than FM-RME [158,159,160,161].

Cevidanes et al. conducted a study to compare the treatment of patients with Class III dento-skeletal malocclusion with Bone-Anchored Maxillary Protraction (BAMP) and with Face Mask-Rapid Maxillary Expander (FM-RME). The results showed that BAMP resulted in significantly greater upper jaw advancement (approximately 2.5–3 mm) than FM-RME, with significant improvements in midface length and molar relationship. Although the sagittal changes to the mandible were similar, BAMP better controlled vertical growth and avoided retroclination of the lower incisors. However, the BAMP treatment requires surgery for insertion and removal of the anchors, whereas the FM-RME protocol is less invasive but requires more time to use the device [162,163,164,165].

Gencer et al. conducted a retrospective study to compare two types of orthodontic treatment of patients with Class III dento-skeletal malocclusion and anterior crossbite. One group of patients was managed with a Delaire-type Face Mask (FM), while the other group was managed with a Face Mask (FM) and an Anterior Protrusion Device (DPA). Both treatments improved the molar relationship and over-jet, but DPA-FM reduced the undesired effect of mandibular post-rotation [166,167,168].

### 3.2. Elastodontic Therapy

These findings are consistent with previous studies reporting that elastodontic appliances may contribute to improvements in sagittal relationships and neuromuscular balance in growing patients. Elastodontic therapy is an evolution of functional orthodontics [169,170,171].

Elastodontics was introduced in Europe to treat malocclusions in mixed dentition patients [172,173,174]. Elastodontic therapy uses mild elastic forces to produce both skeletal and dental effects [175,176,177]. It is also called myofunctional therapy because it restores muscle function by neutralising centrifugal and centripetal forces [178,179,180,181]. Treatment with elastodontic devices restores nasal breathing, lip sealing, correct lingual posture and physiological swallowing [182,183,184]. It is necessary to educate the patient to perform certain exercises at home to correct lingual, masticatory and respiratory functions [185,186,187]. These effects are mainly related to the neuromuscular action of the appliance. The vestibular flange acts as a lip bumper, reducing the pressure of the orbicularis and buccinator muscles and promoting passive lip sealing. The lingual ramp and tongue button guide the tongue toward a more physiological palatal position, encouraging nasal breathing and reducing the tendency for low tongue posture. In addition, the presence of the appliance during swallowing helps reprogram the oro-facial muscles, promoting a more physiological swallowing pattern by discouraging anterior tongue thrust and facilitating correct tongue-palate contact. Because the study involved growing patients, part of the observed cephalometric changes may also reflect physiological craniofacial growth rather than treatment effects alone.

There are different types of appliances depending on the malocclusion to be treated; in fact, they are preformed appliances, but there are auxiliary elements that can be added if necessary [188,189,190].

Regarding the material used for their construction, soft silicones or polymeric compositions are generally used to generate light elastic forces that do not traumatise the oral mucosa [191,192,193,194].

The ideal time for elastodontic therapy is in the primary or mixed dentition to achieve orthopaedic and orthodontic effects [195,196,197]. These devices can be used not only in the interceptive phase in conjunction with myofunctional exercises, but also in the restraint phase to maintain the results obtained over time [198,199,200,201].

Commercially available elastodontic appliances include [202,203,204,205]:

- EF devices designed by Daniel Rollet (Éducation Fonctionnelle, Orthoplus^®^, Igny, France);
- T4K and POT (Trainer For Kids e Pre-Orthodontic Trainer), actually called Myobrace^®^ (MRC Myofunctional Research, Helensvale, Australia);
- LM-Activator (LM, Parainen, Finland);
- MFS (Multi-Function System) by Duran von Arx (Orthodontic World, Barcelona, Spain);
- Muppy^®^ Oral Screen developed by Hintz (DHD, Herne, Germany);
- Occlus-o-Guide^®^, Nite-Guide^®^, Class III^®^ and Healthy Start™ (Sweden and Martina);
- AMCOP^®^ (Multifunctional Cranio-Occlusal-Postural Harmonisers) by Micerium.

The AMCOP^®^ bio-activators are functional devices with neuromuscular, orthopaedic and orthodontic effects [206,207,208,209]. These devices are indicated in patients of developmental age; in fact, the plasticity and adaptability of skeletal structures allow the removal of factors responsible for malocclusion [210,211,212]. In addition, AMCOP^®^ are well tolerated by patients and require simple handling and cooperation [213,214,215,216]. After a treatment of 16–20 months with AMCOP^®^ bio-activators, there is a significant radiographic and clinical improvement in the skeletal and dento-alveolar situation of patients [217,218,219].

A review by Ureni et al. examined, by means of cephalometric measurements, the effectiveness of elastodontic devices in the treatment of children with Class II and Class III dento-skeletal malocclusions, comparing them with patients treated with conventional orthodontic appliances. The results showed that elastodontic therapy is able to bring about improvements in sagittal relationships and overall tooth alignment, especially in patients with altered oral habits such as atypical swallowing. However, such devices require patient compliance and would appear to be less effective than conventional orthodontic appliances in bringing about skeletal and soft tissue effects [220,221,222,223].

In a study by Ciavarella et al., patients with advanced mixed dentition and Class II malocclusion were treated with an elastodontic device. After 24 months, significant improvements were found in mandibular length and lower facial height, but no significant changes in tooth inclinations or aesthetic variables. In conclusion, the elastodontic appliance showed limited effects on the correction of malocclusion, mainly affecting mandibular growth [224,225,226].

Ronsivalle et al. performed a retrospective study to compare the treatment of patients with Class III dento-skeletal malocclusion with bi-maxillary plates and Class III elastodontic appliances and with a one-piece elastodontic appliance. The authors concluded that both types of therapy result in significant improvement, but elastodontics provide greater transverse palatal growth [227,228,229,230]. However, the retrospective design and the limited sample may introduce selection bias.

## 4. Case Series Presentation

Individual cephalometric measurements for each patient are reported in the corresponding case tables. Given the limited sample size, the results are presented descriptively without inferential statistical testing.

### 4.1. Clinical Case #1

**Figure 3:**
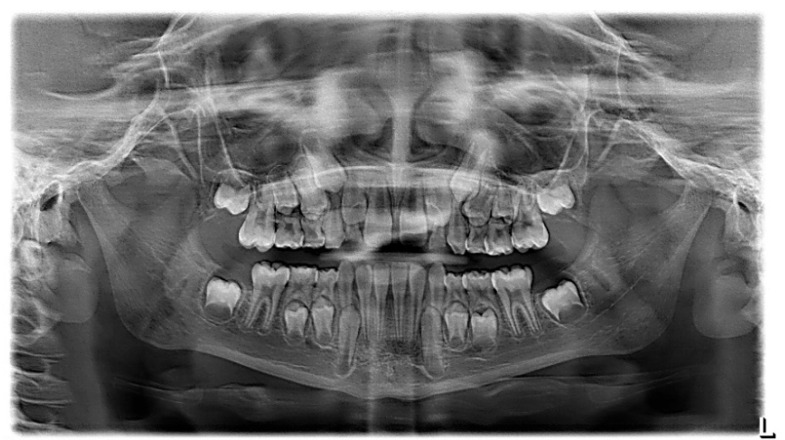
Orthopantomography X-ray before treatment (clinical case #1).

**Figure 4:**
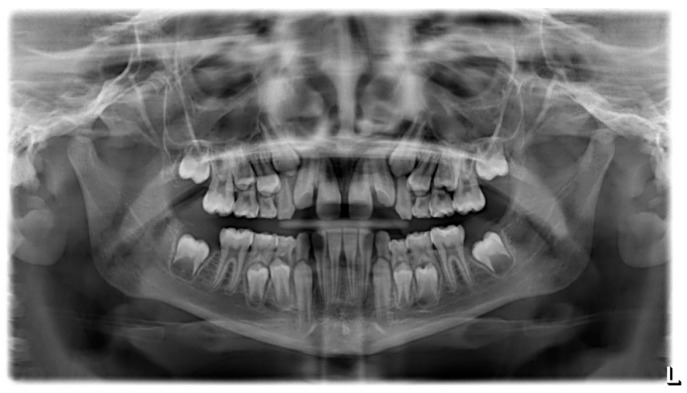
Orthopantomography X-ray after the treatment (clinical case #1).

**Figure 5:**
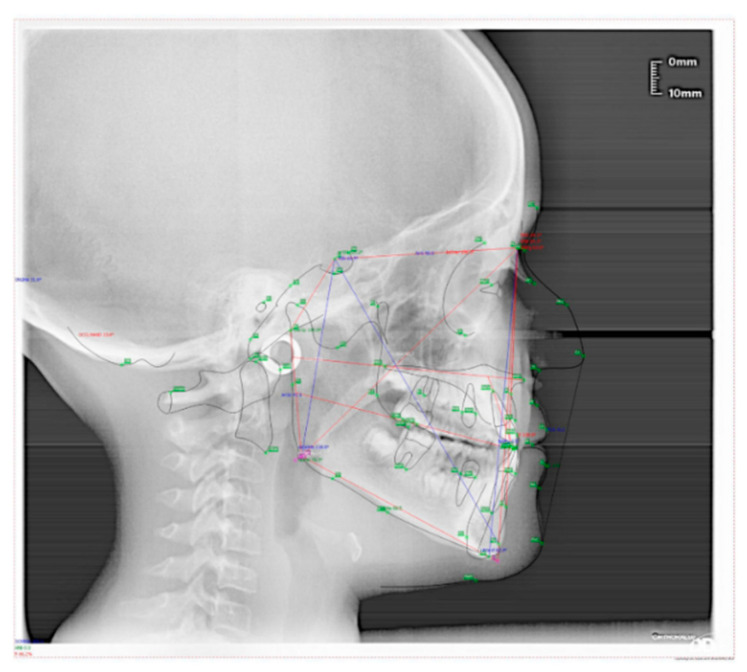
Cephalometric tracing (DeltaDent software) before treatment reveals a skeletal Class III (clinical case #1).

**Figure 6:**
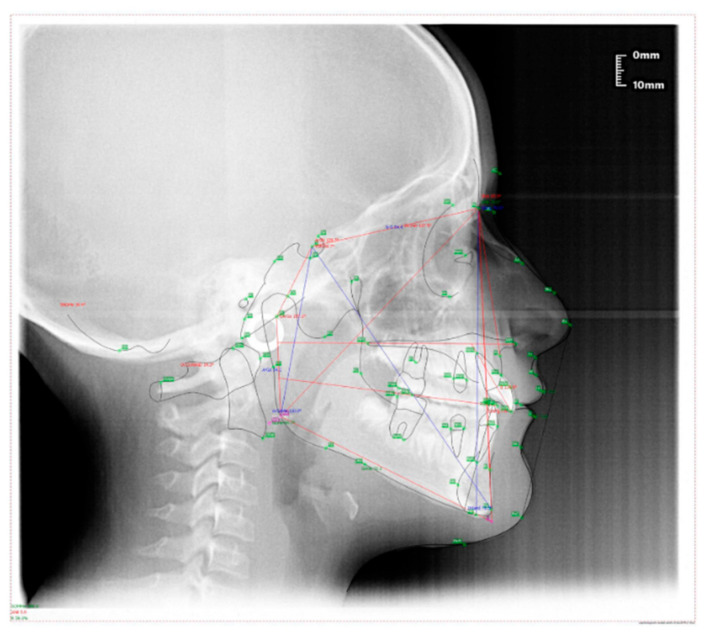
Cephalometric tracing (DeltaDent software) after the treatment (clinical case #1).

**Figure 7:**
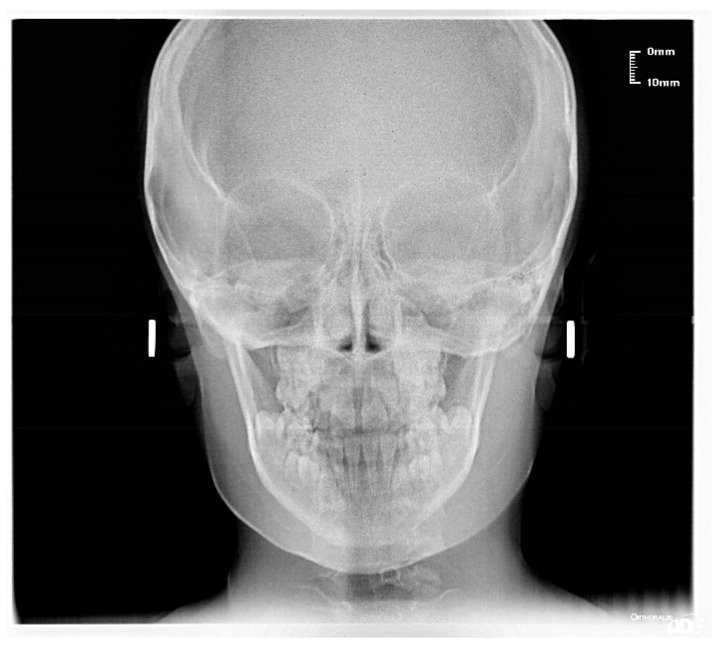
Postero-anterior Teleradiography before treatment (clinical case #1).

**Figure 8:**
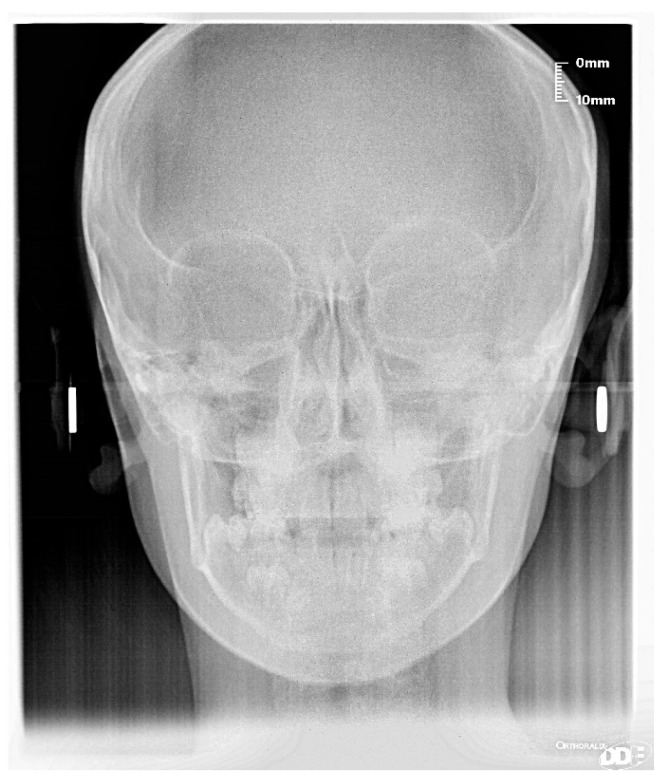
Postero-anterior Teleradiography after the treatment (clinical case #1).

**Figure 9:**
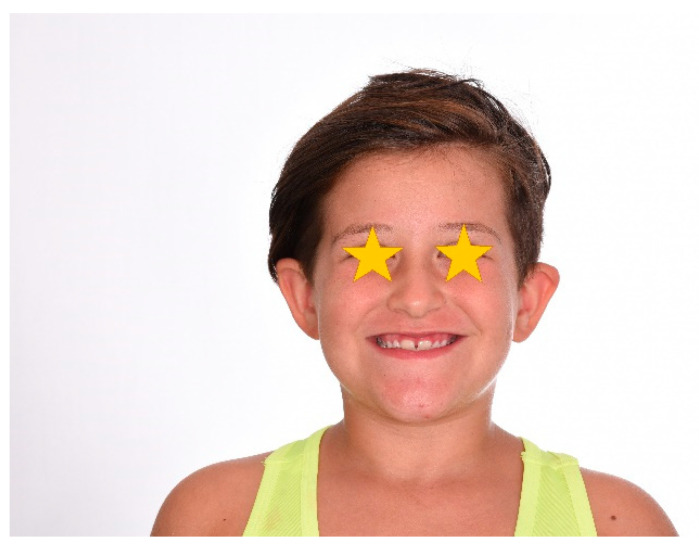
Initial extraoral photograph (clinical case #1).

**Figure 10:**
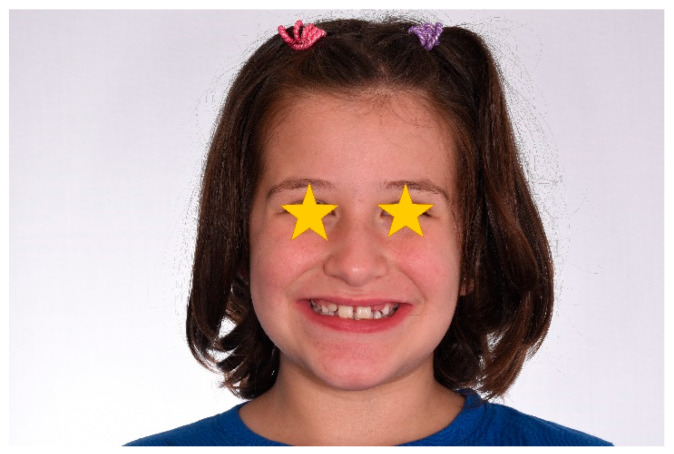
Extraoral photograph after the treatment (clinical case #1).

**Figure 11:**
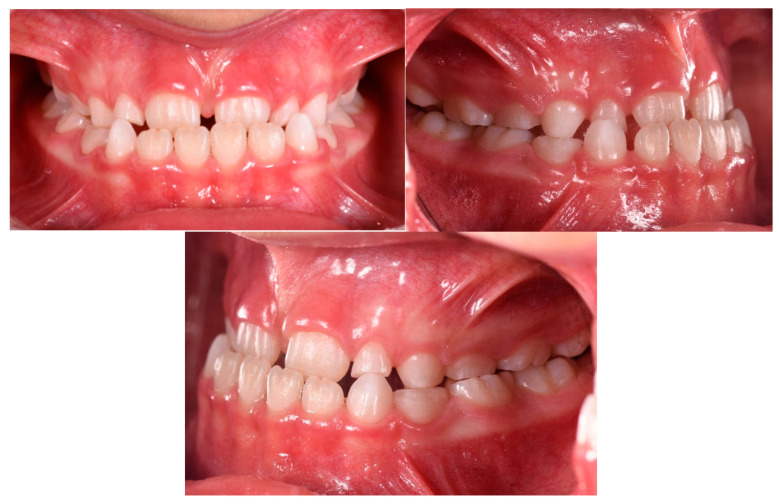
Initial intraoral photographs (frontal view and lateral view) of the subject (7-year-old) (clinical case #1).

**Figure 12:**
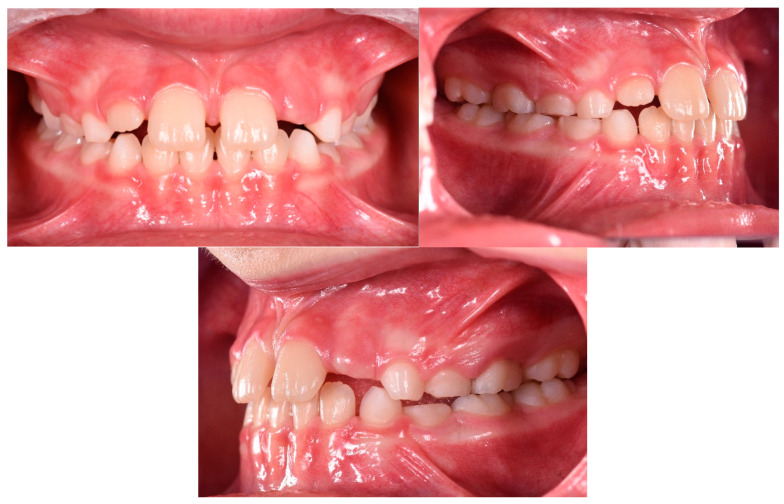
Intraoral photographs (frontal view and lateral view) after the treatment (clinical case #1).

The patient was a 7-year-old boy with a Class III dento-skeletal malocclusion treated with AMCOP^®^ TC. The medical history revealed respiratory difficulties and recurrent colds. Before and after the treatment, orthopantomography (Figure 3 and Figure 4), latero-lateral teleradiography (Figure 5 and Figure 6), postero-anterior teleradiography (Figure 7 and Figure 8), cephalometric examination (Table 1 and Table 2), extraoral photographs (Figure 9 and Figure 10) and intraoral photographs (Figure 11 and Figure 12) were performed.

Extraoral examination revealed an anterior crossbite, interincisive diastema, contraction of the maxillary arch, and insufficient space for the correct eruption of 1.2 and 2.2. The pre-treatment cephalometric analysis (Deltadent^®^ Lana, Bolzano, Italy) revealed a skeletal Class III. The proposed treatment plan included the use of the AMCOP^®^ TC appliance. The patient wore the device for two hours in the afternoon and all night for 6 months and only at night for a further 6 months. At the end of the treatment, the patient had a Class I dentition with correction of overjet and overbite; the device also allowed tongue reeducation and postural restoration of the first cervical vertebrae.

### 4.2. Clinical Case #2

**Figure 13:**
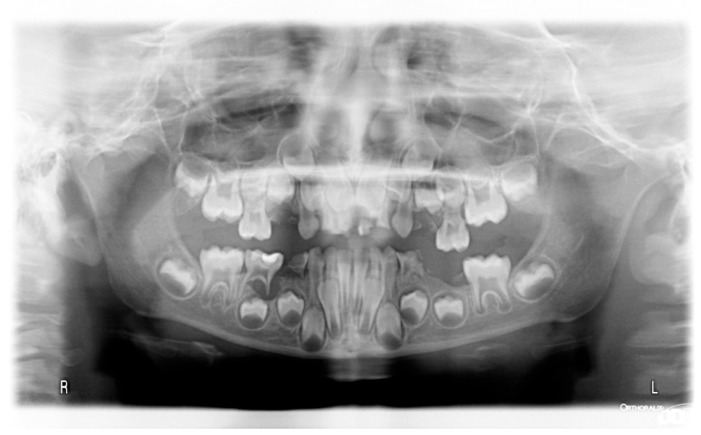
Orthopantomography X-ray before treatment (clinical case #2).

**Figure 14:**
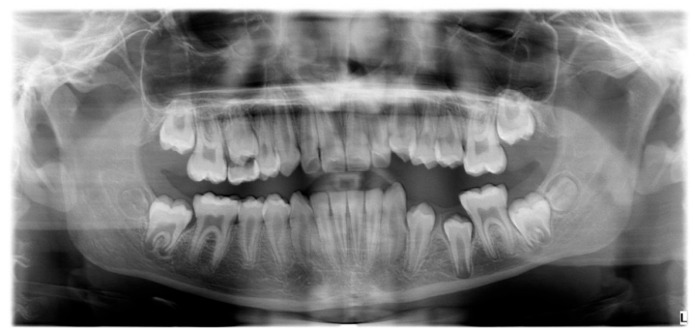
Orthopantomography X-ray after the treatment (clinical case #2).

**Figure 15:**
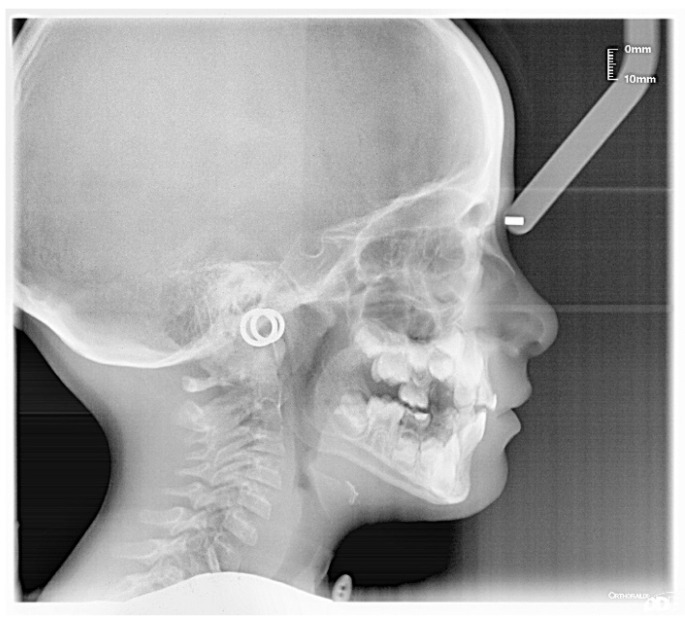
Latero-lateral Teleradiography before treatment (clinical case #2).

**Figure 16:**
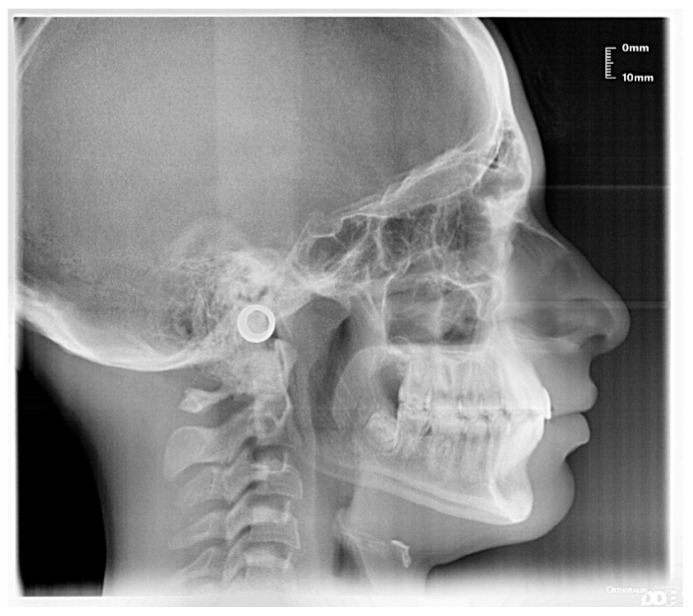
Latero-lateral Teleradiography after the treatment (clinical case #2).

**Figure 17:**
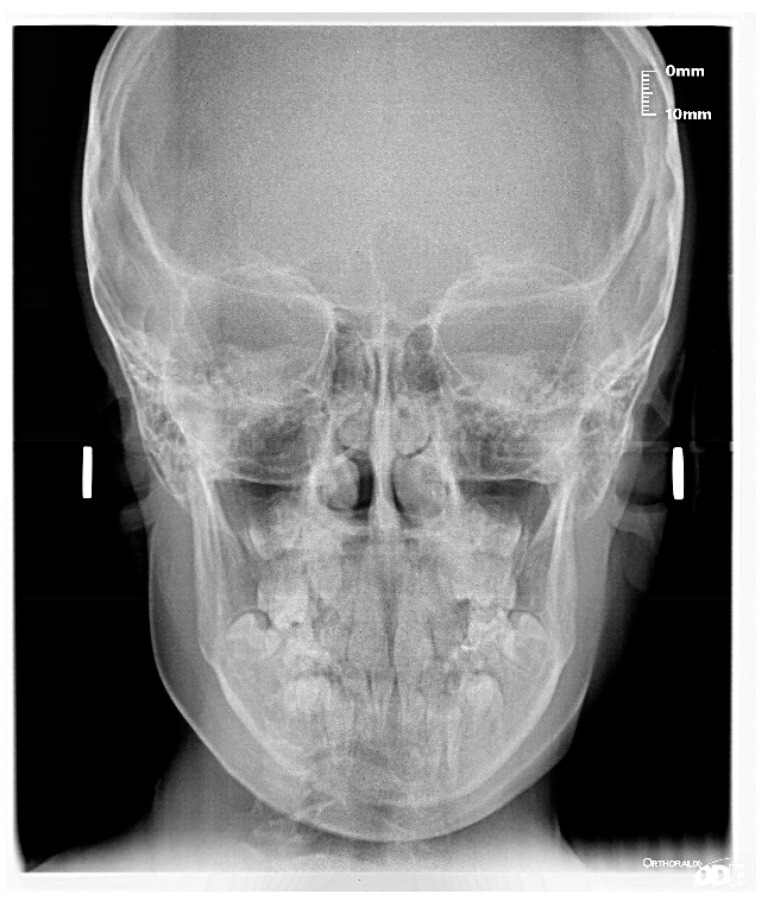
Postero-anterior Teleradiography before treatment (clinical case #2).

**Figure 18:**
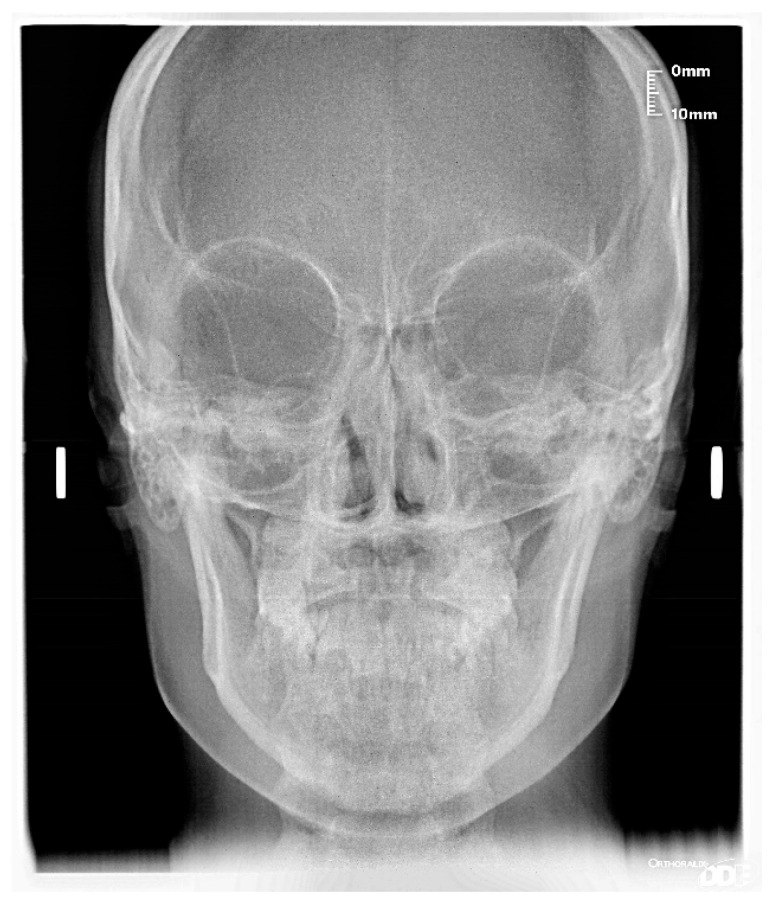
Postero-anterior Teleradiography after the treatment (clinical case #2).

**Figure 19:**
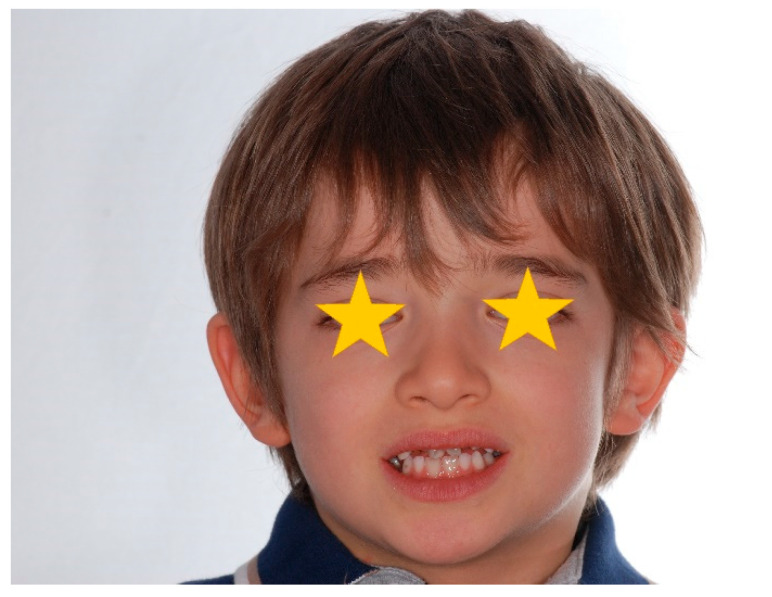
Initial extraoral photograph (clinical case #2).

**Figure 20:**
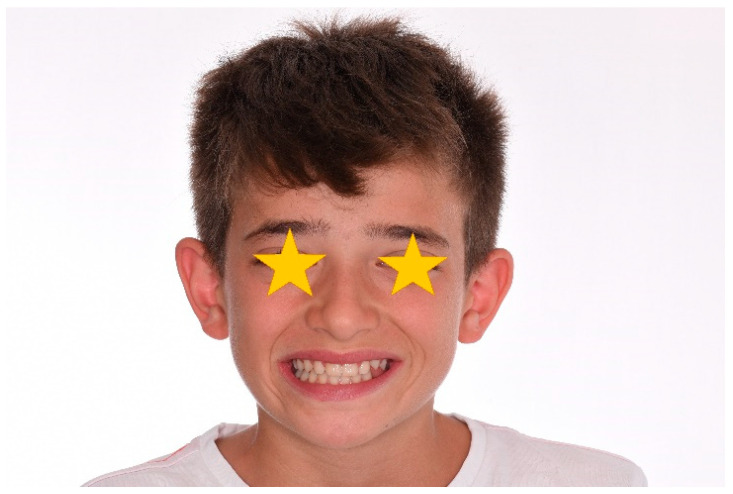
Extraoral photograph after the treatment (clinical case #2).

**Figure 21:**
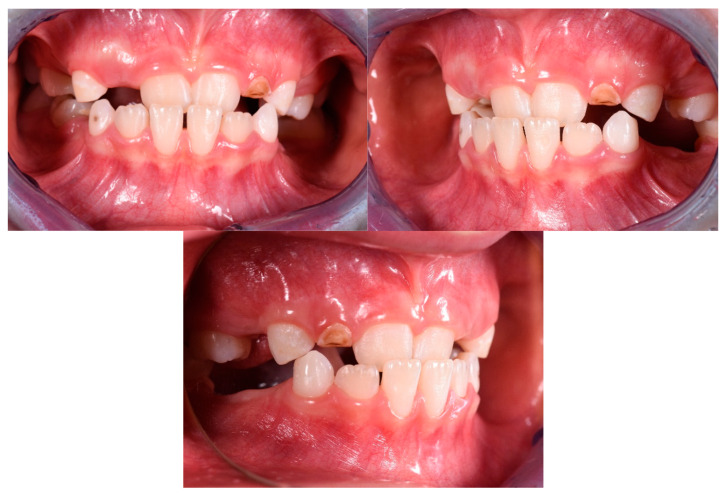
Initial intraoral photographs (frontal view and lateral view) of the subject (7-year-old) (clinical case #2).

**Figure 22:**
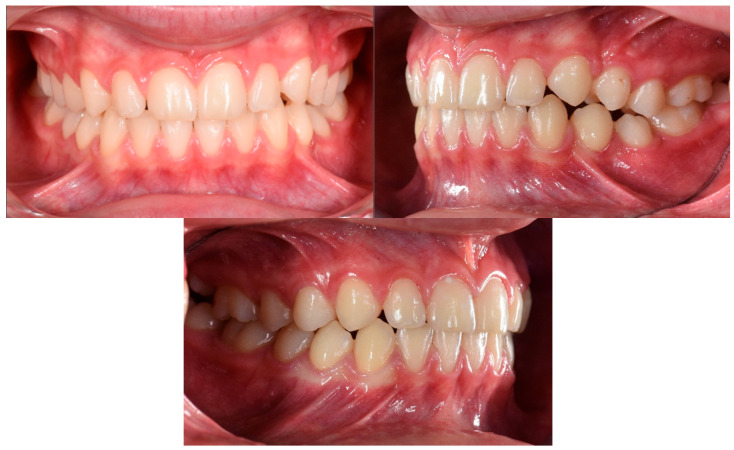
Intraoral photographs (frontal view and lateral view) after the treatment (clinical case #2).

The patient was a 7-year-old boy with a Class III dento-skeletal malocclusion treated with AMCOP^®^ TC. The medical history revealed respiratory difficulties and recurrent colds. Before and after the treatment, orthopantomography (Figure 13 and Figure 14), latero-lateral teleradiography (Figure 15 and Figure 16), postero-anterior teleradiography (Figure 17 and Figure 18), cephalometric examination (Table 3 and Table 4), extraoral photographs (Figure 19 and Figure 20) and intraoral photographs (Figure 21 and Figure 22) were performed.

Extraoral examination revealed an anterior crossbite, interincisive diastema, contraction of the maxillary arch, and insufficient space for the correct eruption of 1.2 and 2.2. The pre-treatment cephalometric analysis (Deltadent^®^ Lana, Bolzano, Italy) revealed a skeletal Class III. The proposed treatment plan included the use of the AMCOP^®^ TC appliance. The patient wore the device for two hours in the afternoon and all night for 6 months and only at night for a further 8 months. At the end of the treatment, the patient had a dento-skeletal Class I and corrected overjet and overbite; the appliance also allowed tongue reeducation and postural recovery of the first cervical vertebrae.

### 4.3. Clinical Case #3

**Figure 23:**
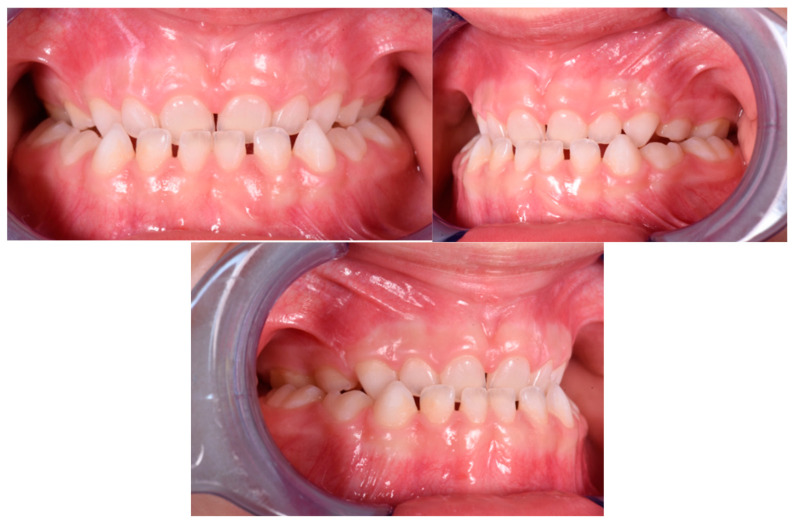
Initial intraoral photographs (frontal view and lateral view) of the subject (3-year-old) (clinical case #3).

**Figure 24:**
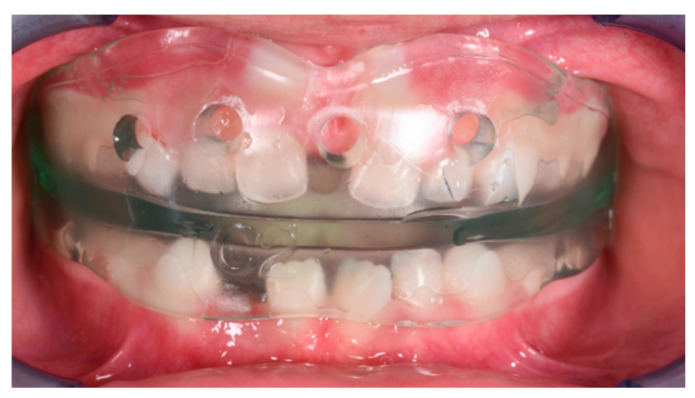
Intraoral photograph with the AMCOP^®^ TC (clinical case #3).

**Figure 25:**
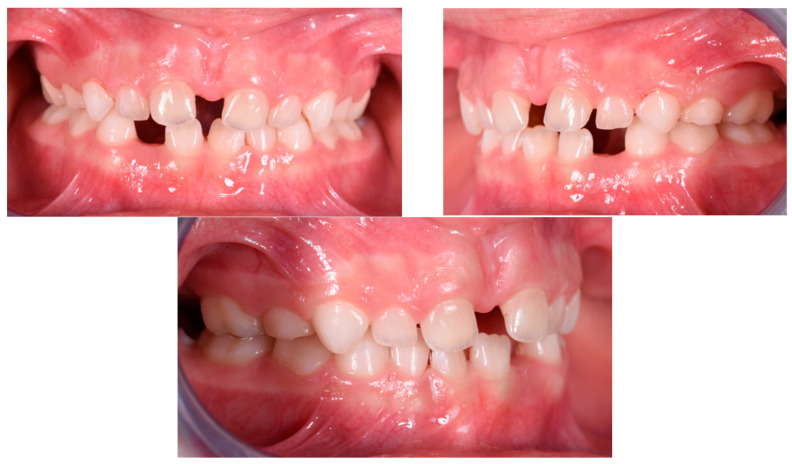
Intraoral photographs (frontal view and lateral view) after the treatment (clinical case #3).

The patient was a 3-year-old female with a Class III dento-skeletal malocclusion treated with AMCOP^®^ TC. The medical history revealed the presence of this type of malocclusion in the parents. The extraoral examination revealed an anterior crossbite and a Class III molar and canine on the right and left (Figure 23). The proposed treatment plan involved the use of the AMCOP^®^ TC device (Figure 24). The patient wore the device for two hours in the afternoon and all night for 6 months and only at night for a further 10 months. At the end of the treatment, the patient had a Class I dentoskeleton with a corrected overjet and overbite (Figure 25). To stabilise the result achieved and to guide the eruption of the permanent teeth, the patient wore the appliance only overnight. The patient was followed up with six-monthly visits.

### 4.4. Clinical Case #4

**Figure 26:**
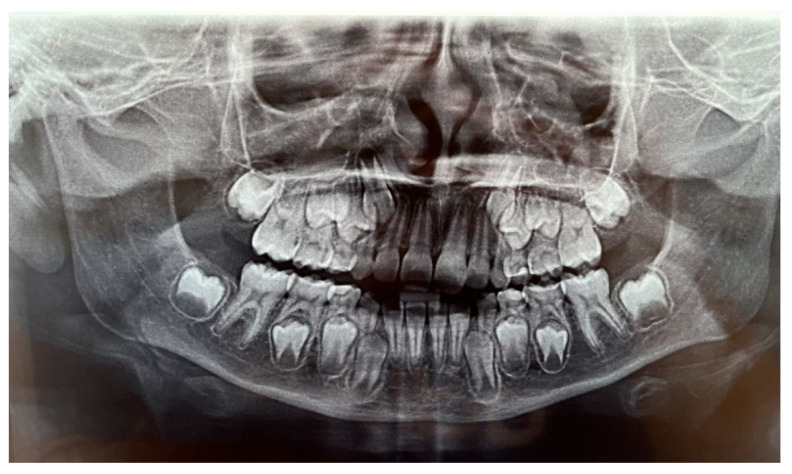
Orthopantomography X-ray before treatment (clinical case #4).

**Figure 27:**
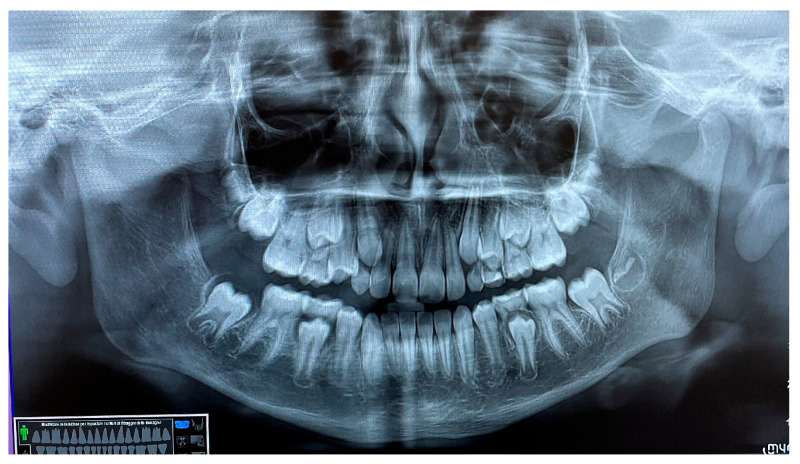
Orthopantomography X-ray after the treatment (clinical case #4).

**Figure 28:**
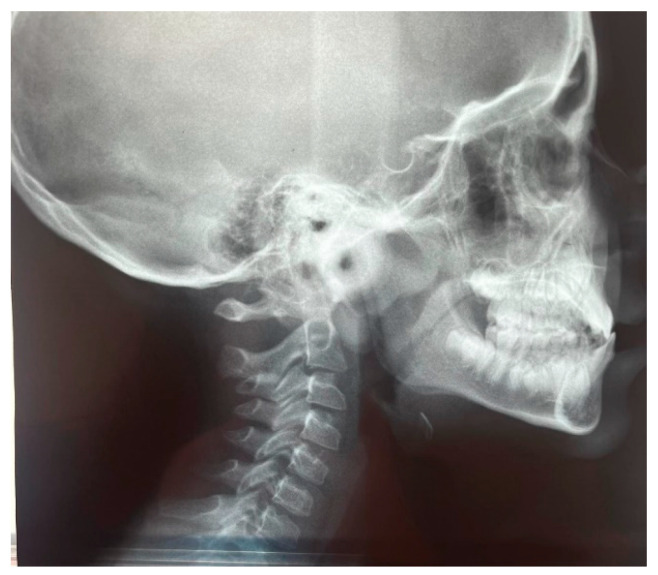
Latero-lateral Teleradiography before treatment (clinical case #4).

**Figure 29:**
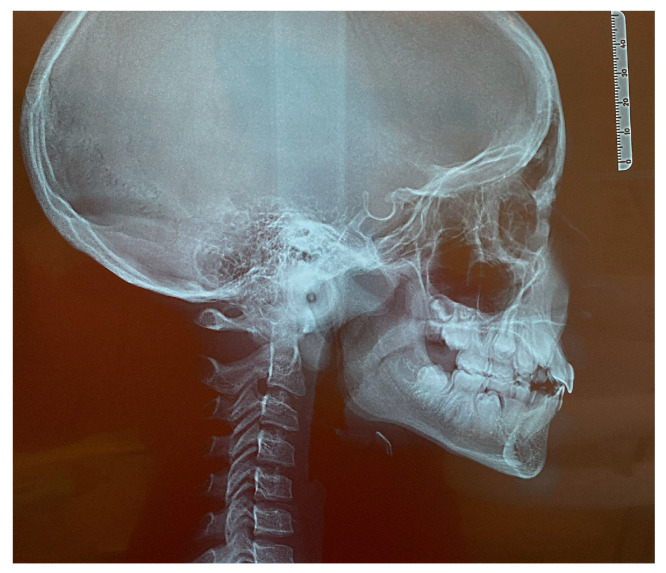
Latero-lateral Teleradiography after the treatment (clinical case #4).

**Figure 30:**
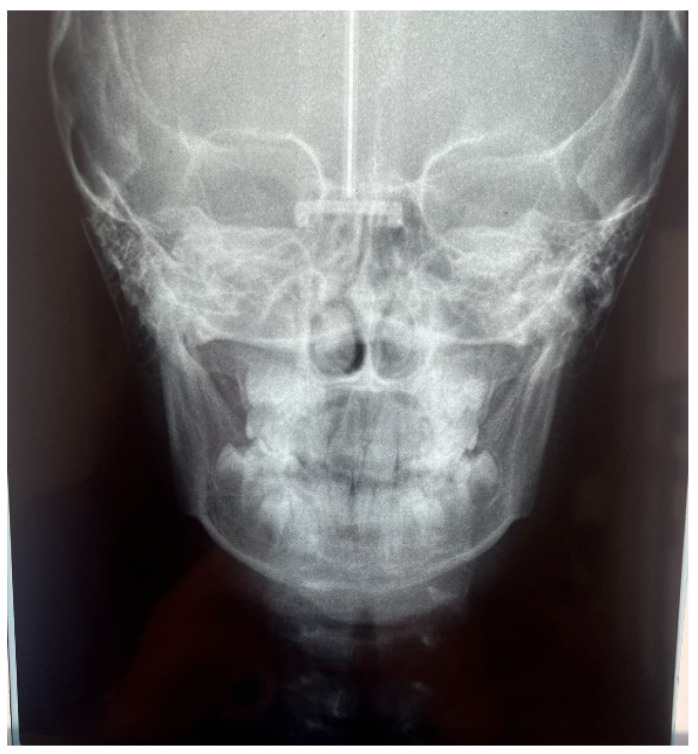
Postero-anterior Teleradiography before treatment (clinical case #4).

**Figure 31:**
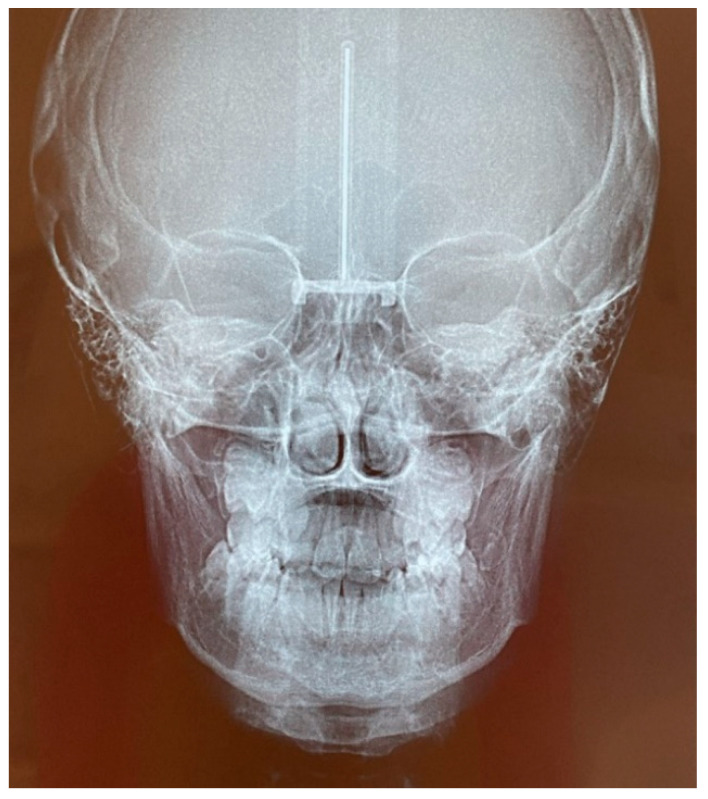
Postero-anterior Teleradiography after the treatment (clinical case #4).

**Figure 32:**
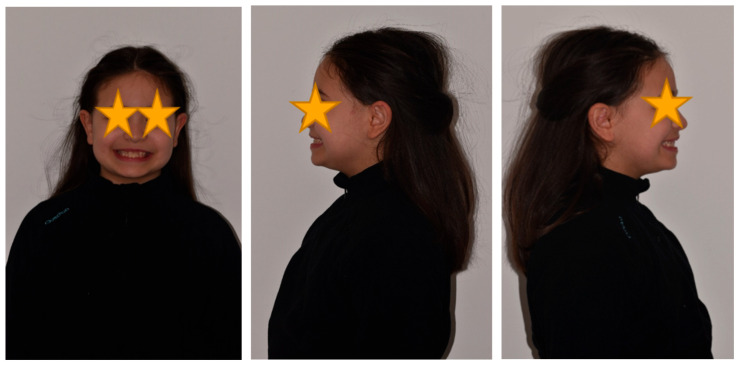
Initial extraoral photographs (clinical case #4).

**Figure 33:**
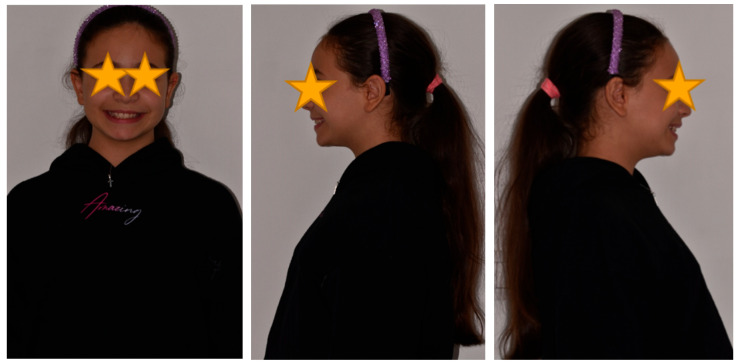
Extraoral photographs after the treatment (clinical case #4).

**Figure 34:**
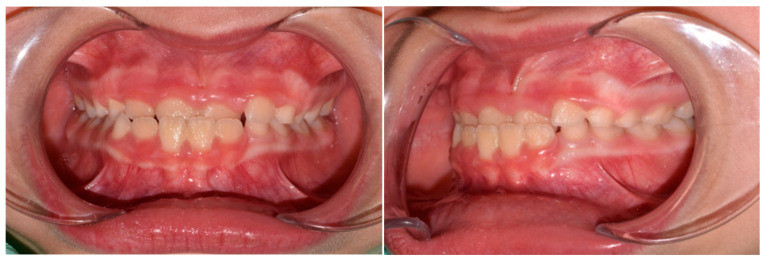

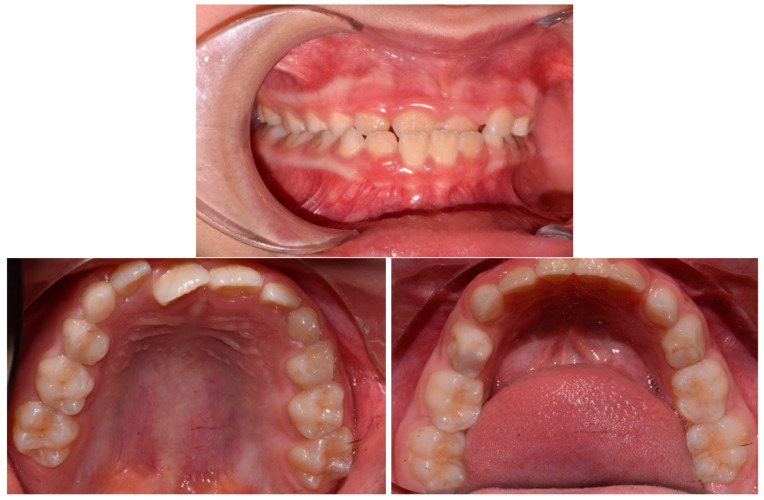
Initial intraoral photographs (clinical case #4).

**Figure 35:**
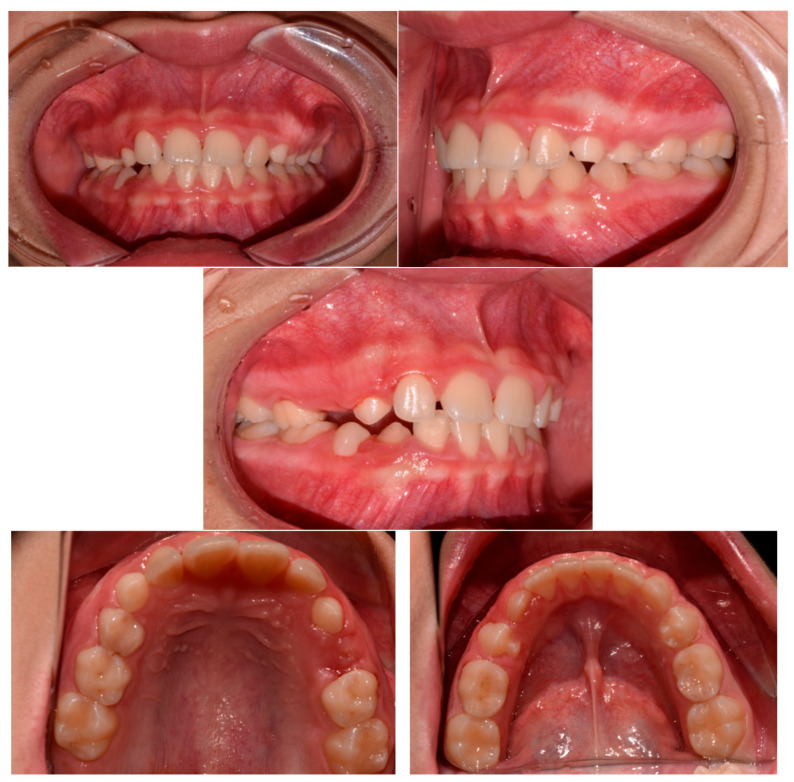
Intraoral photographs after the treatment (clinical case #4).

The patient was a 10-year-old female with a Class III dento-skeletal malocclusion treated with AMCOP^®^ TC. Before and after the treatment, orthopantomography (Figure 26 and Figure 27), latero-lateral teleradiography (Figure 28 and Figure 29), postero-anterior teleradiography (Figure 30 and Figure 31), cephalometric examination (Table 5, Table 6, Table 7, Table 8, Table 9 and Table 10), extraoral photographs (Figure 32 and Figure 33), and intraoral photographs (Figure 34 and Figure 35) were performed. At the end of the treatment, the patient’s dental impressions were also taken. The proposed treatment plan included the use of the AMCOP^®^ TC device. The patient wore the appliance for two hours in the afternoon and all night for 6–8 months and then only at night for the following months; the treatment period was 1 year and 7 months. At the end of the treatment, the patient showed resolution of the Class III dento-skeletal malocclusion, as well as a measurable improvement in posture at the level of the first cervical vertebrae.

This study has several limitations, in particular its retrospective design, small sample size, absence of a control group, variable follow-up, growth-related changes, and non-blinded measurements.

## 5. Conclusions

The clinical cases reported functional therapy with the AMCOP^®^ TC elastodontic device, worn by the patients for two hours in the afternoon and all night for 6–8 months, and then only at night for the following months, was associated with improvement of the Class III dento-skeletal relationship and postural recovery of the first cervical vertebrae. All treatments had an average duration of 16–24 months, and two or a maximum of three AMCOP^®^ were changed because every 6–8 months the appliance was changed because it lost elasticity. Long-term follow-up studies are needed to evaluate the stability of the obtained results and the potential risk of relapse.

However, further literature studies are needed regarding the use of AMCOP^®^ TC bio-activators for the treatment of Class III dento-skeletal malocclusions. These devices are minimally invasive and have good compliance, so they may be a viable treatment option. However, due to the retrospective design, the small sample size, and the absence of a control group, the present findings should be interpreted with caution and considered preliminary.

## Figures and Tables

**Table 1:** Cephalometric analysis before treatment (clinical case #1).

Cephalometric Analysis Before Treatment	Values (°)	Normal Values (°)
SNA	84	82 ± 3
SNB	84	80 ± 3
ANB	0	2 ± 2
S-Ar-Go	145	143 ± 6
Ar-Go-Me	118	130 ± 7
−1 GoMe	82	94 ± 7
+1 SN	106	102 ± 2
II	139	132 ± 6

**Table 2:** Cephalometric Analysis after Treatment (Clinical Case #1).

Cephalometric Analysis After Treatment	Values (°)	Normal Values (°)
SNA	85	82 ± 3
SNB	81	80 ± 3
ANB	4	2 ± 2
S-Ar-Go	150	143 ± 6
Ar-Go-Me	110	130 ± 7
−1 GoMe	80	94 ± 7
+1 SN	107	102 ± 2
II	135	132 ± 6

**Table 3:** Cephalometric analysis before treatment (clinical case #2).

Cephalometric Analysis Before Treatment	Values (°)	Normal Values (°)
SNA	84	82 ± 3
SNB	84	80 ± 3
ANB	0	2 ± 2
S-Ar-Go	145	143 ± 6
Ar-Go-Me	118	130 ± 7
−1 GoMe	82	94 ± 7
+1 SN	106	102 ± 2
II	139	132 ± 6

**Table 4:** Cephalometric Analysis after Treatment (clinical case #2).

Cephalometric Analysis After Treatment	Values (°)	Normal Values (°)
SNA	85	82 ± 3
SNB	81	80 ± 3
ANB	4	2 ± 2
S-Ar-Go	150	143 ± 6
Ar-Go-Me	110	130 ± 7
−1 GoMe	80	94 ± 7
+1 SN	107	102 ± 2
II	135	132 ± 6

**Table 5:** Jaraback Cephalometric analysis before treatment (clinical case #4).

Jaraback Before Treatment	Values (°)	Normal Values (°)
SNA	89	82 ± 3
SNB	88	80 ± 3
ANB	1	2 ± 2
SN-GoGn	24	32 ± 3
N-S-Ar	126	123 ± 5
S-Ar-Go	127	143 ± 6
Ar-Go-Gn	131	130 ± 7
Total	384	396 ± 6
Anterior Cranial Base	56	71 mm ± 3
Cranial Base Posterior	28	32 mm ± 3
Upper Goniac Corner	59	52–55
Lower Goniac Corner	72	70–75
Branch Length	45	44 mm ± 5
Body Length	63	71 mm ± 5
Anterior Cranial Base/Mandibular Body Ratio	7	1/1
Rear Facial Height	65	70–85 mm
Front Facial Height	88	105–120 mm
Front/Rear Facial Height	0.7	62–65%
Ui-Li	123	132 ± 6
Upper Facial Incisor	2	5 mm ± 2
Lower Incisor to Facial Plane	3	2 mm ± 2

**Table 6:** Jaraback Cephalometric analysis after the treatment (clinical case #4).

Jaraback After Treatment	Values (°)	Normal Values (°)
SNA	90	82 ± 3
SNB	85	80 ± 3
ANB	5	2 ± 2
SN-GoGn	25	32 ± 3
N-S-Ar	128	123 ± 5
S-Ar-Go	131	143 ± 6
Ar-Go-Gn	125	130 ± 7
Total	384	396 ± 6
Anterior Cranial Base	59	71 mm ± 3
Cranial Base Posterior	30	32 mm ± 3
Upper Goniac Corner	56	52–55
Lower Goniac Corner	69	70–75
Branch Length	44	44 mm ± 5
Body Length	67	71 mm ± 5
Anterior Cranial Base/Mandibular Body Ratio	8	1/1
Rear Facial Height	68	70–85 mm
Front Facial Height	94	105–120 mm
Front/Rear Facial Height	0.7	62–65%
Ui-Li	116	132 ± 6
Upper Facial Incisor	7	5 mm ± 2
Lower Incisor to Facial Plane	4	2 mm ± 2

**Table 7:** EBO Cephalometric analysis before treatment (clinical case #4).

EBO Before Treatment	Values (°)	Normal Values (°)
Sagittal bone relations		
SN/A	88.6	82 ± 3.5
SN/Pg	88.6	80 ± 3.5
AN/Pg	0	2 ± 2.5
Vertical bone relations		
SN/AnsPns	4.8	8 ± 3
SN/GoGn	24.3	33 ± 2.5
AnsPns/GoGn	19.5	25 ± 6
Dento-basal relations		
+ 1/AnsPns	113.7	110 ± 6
−1 GoGn	103.9	94 ± 7
−1 A/Pog	3.0	2 mm ± 2
Dental reports		
Overjet	−1.7	3.5 mm ± 2.5
Overbite	1	2.5 mm ± 2.5
Inter-incisal angle	122.9	132 ± 6

**Table 8:** EBO Cephalometric analysis after the treatment (clinical case #4).

EBO After Treatment	Values (°)	Normal Values (°)
Sagittal bone relations		
SN/A	89.9	82 ± 3.5
SN/Pg	85.2	80 ± 3.5
AN/Pg	4.7	2 ± 2.5
Vertical bone relations		
SN/AnsPns	3.5	8 ± 3
SN/GoGn	25	33 ± 2.5
AnsPns/GoGn	21.6	25 ± 6
Dento-basal relations		
+ 1/AnsPns	114.1	110 ± 6
−1 GoGn	108.6	94 ± 7
−1 A/Pog	2.0	2 mm ± 2
Dental reports		
Overjet	2.7	3.5 mm ± 2.5
Overbite	1.6	2.5 mm ± 2.5
Inter-incisal angle	115.7	132 ± 6

**Table 9:** Giannì Cephalometric analysis before treatment (clinical case #4).

Giannì Before Treatment	Values (°)	Normal Values (°)
SNA	93	80–84
SNB	92	78–82
ANB	1	0–4
Saddle corner	119	124–134
Articular angle	140	137–149
Upper gonian angle	54	48–52
Lower goniac angle	69	68–72
Total goniac angle	123	115–125
Total	382	390–400
Angle of divergence	22	29–35
Upper incisal inclination	114	100–104
Lower incisal inclination	103	85–95
Inter-incisor angle	121	126–136
Anterior skull base	54.73 mm	71 mm (at the age of 11)
Posterior skull base	26.07 mm	29–35 mm
Length of mandibular body	56.67 mm	71 mm

**Table 10:** Giannì Cephalometric analysis after the treatment (clinical case #4).

Giannì After Treatment	Values (°)	Normal Values (°)
SNA	89	80–84
SNB	86	78–82
ANB	3	0–4
Saddle corner	130	124–134
Articular angle	135	137–149
Upper gonian angle	53	48–52
Lower goniac angle	68	68–72
Total goniac angle	121	115–125
Total	386	390–400
Angle of divergence	25	29–35
Upper incisal inclination	110	100–104
Lower incisal inclination	105	85–95
Inter-incisor angle	120	126–136
Anterior skull base	58.80 mm	71 mm (at the age of 11)
Posterior skull base	32.01 mm	29–35 mm
Length of mandibular body	64.38 mm	71 mm

## Data Availability

Anonymized data are available from the corresponding author upon reasonable request. The data are not publicly available due to privacy and ethical restrictions.

## Abbreviations

AMCOP	Multifunctional Cranio-Occlusal-Postural Harmoniser
BAMP	Bone Anchored Maxillary Protraction
DPA	Anterior Protrusion Device
EF	Éducation Fonctionnelle
FM	Face Mask
FM-RME	Face Mask–Rapid Maxillary Expander
MFS	Multi-Function System
MRC	Myofunctional Research
POT	Pre-Orthodontic Trainer
RPE	Rapid Palatal Expander
RTBLP-RME	Reverse Twin Block with Lip Pads–Rapid Maxillary Expander
SC	Second Class
T4K	Trainer For Kids
TC	Third Class
TSME	Transverse Saggital Maxillary Expander
